# Associations of Food Outlet Densities with Obesity Measures Identify Fish and Chip Shops as a Uniquely Important Problem

**DOI:** 10.3390/nu12040890

**Published:** 2020-03-25

**Authors:** Ahmad Albalawi, Catherine Hambly, John Speakman

**Affiliations:** 1 School of Biological Sciences, University of Aberdeen, Tillydrone Ave, Aberdeen AB24 2TZ, UK; r03aaaa@abdn.ac.uk (A.A.); c.hambly@abdn.ac.uk (C.H.); 2 Institute of Genetics and Development Biology, Chinese Academy of Sciences, Beijing 100190, China; 3 Centre of Excellence in Animal Evolution and Genetics, Chinese Academy of Sciences, Kunming 650223, Yunnan, China

**Keywords:** obesity, food outlets, fast-food restaurants, full-service restaurants, pubs, cafes, takeaways, food delivery, and fish and chip shops

## Abstract

Increases in the number of food outlets have been proposed as a key factor driving obesity. This study aimed to investigate the association between the densities of food establishments serving meals (excluding supermarkets and grocery stores), with body mass index (BMI), waist to hip ratio (WHR) and percentage of body fat among middle-aged adults in the UK. BMIs, WHR, %fat and socioeconomic factors were obtained from 456,079 individuals from the UK Biobank and averaged across 923 postcode districts (PD). The number of Fast-Food Restaurants (FFRs), Full-Service Restaurants (FSRs), delivery shops, takeaways, fish and chip shops, pubs and cafes were also obtained for each PD. We adjusted the obesity measures for deprivation level, education, employment, ethnicity, household size, household income and age. After adjustment, the density of fish and chip shops (per 1000 population) was positively associated with BMI and %fat for both sexes (males: BMI converted (exponentiated coefficient) β = 0.5, R^2^ = 4.14%, *p* < 0.0001; %fat converted β = 0.8, R^2^ = 3.32%, *p* < 0.0001; females: BMI converted β = 0.9, R^2^ = 5.31%, *p* < 0.0001; %fat converted β = 1.4 R^2^ = 4.65%, *p* < 0.0001). The densities of FFRs and delivery shops (per 1000 population) were not related to the adjusted obesity measures among males and females, except BMI in males where FFRs were significantly negatively associated. The densities (per 1000 population) of FSRs, pubs, cafes and total food outlets were all significantly inversely related to the obesity measures for both sexes. The number of fish and chip shops per 1000 individuals was significantly positively associated with obesity in middle-aged adults in the UK. A negative association between the other types of food outlet densities and the measures of obesity suggests access to such establishments is not a major driver of obesity. This is potentially because the food supplied at such establishments is not significantly less healthy than what is eaten elsewhere including at home (and may even be better). Paying attention only to fast food and/or full-service restaurants in intervention policy will likely not be effective. Policy intervention should potentially focus on the numbers of fish and chip shops and the deep-fried food served in such restaurants.

## 1. Introduction

In the UK, the percentage of adults with obesity in 2016 was 26% [1]. This had risen from 15% in 1993 but had remained at a similar level since 2010—from 25% to 27% [1]. Furthermore, 35.2% of individuals were overweight, making a total of 61.4% either overweight or with obesity [1]. In this regard, the UK is typical of many modern western societies [2]. Expansion in the numbers of Fast-Food Restaurants (FFRs), Full-Service Restaurants (FSRs), takeaways and other types of food outlets that are not supermarkets or groceries, such as pubs and cafes have been proposed as one of the main driving factors for the increased levels of overweight and obesity in the UK [3,4]. Portion sizes in food outlets have increased over time [5]. Out of home meals are substantially larger with more calories and fat and fewer nutrients such as vitamins and minerals [6,7,8]. Moreover, it has been suggested that individuals with lower socioeconomic status are less likely to choose more healthy meals when eating out [9,10,11,12], and this may indicate that there is a relationship between socioeconomic status and obesity [13,14].

Some studies also suggested that there is a significant positive association between the density of fast-food restaurants or takeaways and fast-food consumption and obesity [3,4,5,6,7,8,9,10,11,12,13,14,15,16]. On the other hand, other investigations found there is mot relationship between obesity and increased densities of restaurants once confounding factors such as race, poverty and education were accounted for, but they were linked to elevated rates of cardiovascular mortality and stroke [17,18,19]. The outcomes of the different studies could be due to different methods for accounting for confounding factors. Nevertheless, these studies suggest that the data linking restaurants to being overweight and obese are not strong. The aim of the present study was to investigate the association between the densities of food establishments that serve meals (excluding supermarkets and grocery stores), with body mass index (BMI), waist to hip ratio (WHR) and percentage of body fat among 456,079 middle-aged adults registered in the UK Biobank. The data were analysed unadjusted and adjusted for potential confounding factors.

## 2. Method

### 2.1. Data Sources

Data were obtained on BMI, WHR and %fat and postcode district (PD) for 207,422 males and 248,657 females from the UK Biobank study (project number 41324). The age range that was available on the UK Biobank database was between 40 and 69 years old, who visited one of 22 UK Biobank assessment centres between 2006 and 2010. The UK is divided into PDs.

### 2.2. Inclusion and Exclusion Criteria

Participants who had their obesity measures recorded (BMI, WHR and %fat) and live in PDs that were covered in the Biobank data were included. Districts with less than 25 participants were excluded due to a potentially biased representation. Participants with missing BMI, WHR or %fat measurements were excluded from the study. The total number excluded were 46,461. The total number of eligible PDs with data was 923 out of 2497 across the UK. This is because many PDs lie in areas that are not covered by the UK Biobank study.

The number of FFRs (N = 17,355), FSRs (N = 13,915), fish and chip shops (N = 3617), delivery shops (N = 801), takeaways (N = 3466), pubs (N = 10,610), cafes (N = 9264) and total food outlets (combined) (N = 59,028) in the PDs where the people lived were obtained by compiling data from the Ordnance Survey, Yellow page website and Core List Business Company.

### 2.3. Definition of Food Outlets Densities

The density of each type of food outlet was defined by the number of relevant establishments in each PD per 1000 residents. Delivery shops were defined by the North American Industry Classification System (NAICS) as premises that have services where a customer orders from her/his location and receives the order via food delivery company or personnel from the same restaurant [20]. FFRs were defined by NAICS as establishments that are principally engaged in providing food services where patrons mostly request or select food or drink items and pay before eating [20]. These are separated from ‘takeaways’ because there is the option to consume the ordered food and drinks on the premises. They may however additionally be taken out or delivered to the customer’s residence [20]. Relatively few FFR establishments offer their food services in combination with alcoholic beverage options [20].

FSRs were characterized by NAICS as institutions that principally offer food services where patrons are served by a waitperson while seated [20]. The general pattern is that customers take a seat in the establishment and a waitperson takes their order which is generally then delivered to them where they are seated. An exception to this is establishments where individuals are seated and pay a fixed sum, and food is not delivered to them but rather they self-select their intake from a buffet. Payment at FSR is normally collected after eating [20]. These establishments often also provide alcoholic drinks. They may also provide takeout services [20].

Takeaways were characterized as outlets where meals can be cooked and prepared to be ready for immediate consumption, outside of the establishment [21]. Pubs can be defined as an establishment for selling beer and other alcoholic and non-alcoholic drinks, with food sometimes provided on the premises [21]. The Oxford dictionary defines cafes as small restaurants that can sell small meals and drinks, including tea and coffee as the dominant beverage [22]. Fish and chip shops were defined by the Cambridge dictionary as shops that sell fried battered fish and fried potato chips as the main meal [22]. The food is cooked on the site and can be taken out or served on the premises. Usually, people pay before eating.

### 2.4. Adjustment Variables (Possible Confounding Factors)

Socio-economic factors (deprivation level per household, education, employment, ethnicity, household size, household income and age) were collected for each individual from UK Biobank.

For education level, participants were divided into five categories (O levels/GCSEs which is early secondary school, A levels/AS levels or equivalent which represent late high schools, National Vocational Qualification (NVQ) or higher national diploma (HND) or Higher National Certificate (HNC), Certificate of Secondary Education (CSEs) or equivalent and university or college degree). Household income was divided into five categories (between £18,000 to £30,999; £31,000 to £51,999; £52,000 to £100,000; greater than £100,000 and less than £18,000). The ethnic groups in this study were Caucasians, Chinese, Blacks, Indian, mixed and other races. Moreover, to investigate if these associations are influenced by some types of food outlets, we performed an analysis where the obesity measures further adjusted for fish and chip shops and regressed against the rest of the included food outlets.

### 2.5. Sampling Unit (PDs)

This study used the PD as a sampling unit to aggregate the mean BMI, WHR and %fat data for each PD in comparison with the densities of the different types of food outlets within the same PD. We implemented log transformation for each type of the food outlets per PD and arcsine conversion, where appropriate, for the potential confounding factors (socioeconomic variables) for normalization. The distributions of confounding factors data indicated heteroscedasticity. We attempted different conversions to remove this heteroscedasticity, however, none was effective. The outcome of the analysis was robust to the nature of the data transformations used.

### 2.6. Outcome Variables

The means of BMI and %fat was measured during the visits by health professionals. The equation of BMI is weight in kilograms divided by squared height in meters (Kg/H^2^). Height was measured by utilizing the Seca202 stadiometer and weight and %fat was measured by utilizing the Tanita BC418MA body composition analyser. The hip and waist circumference were obtained from UK Biobank and we divided the waist by hip circumference to calculate WHR for each individual. We then calculated the mean BMI, WHR and %fat for each included PD stratified by sex. The means of BMI, WHR and %fat was used as continuous variables rather than categories of normal, overweight and obese.

### 2.7. Statistical Analysis

We explored the relationship between the unadjusted means of BMI, WHR and %fat for males and females and the food outlet densities using simple linear regressions. The mean BMI, WHR and %fat for both sexes were regressed against all the potential confounding factors as independent factors (deprivation level, education level, employment, ethnicity, household size, household income and age) using multiple linear regressions. We then adjusted the means of BMI, WHR and %fat for the significant confounding factors. The correction of the means of BMI, WHR and %fat was performed by deriving the residuals from the multiple linear regressions for each model and adding the mean BMI, the mean WHR and the mean %fat back to them. These were used as adjusted dependent variables. After correcting the means of BMI, WHR and %fat for the potential confounding factors, the regression analyses were repeated with FFRs, FSRs, delivery shops, takeaways, fish and chip shops, pubs, cafes, the total food outlet densities (combined) and the density of total food outlets per PD per kilometre squared as predictors.

We performed an additional analysis using independent sample t-tests to compare the adjusted BMI, adjusted WHR and the adjusted %fat between PDs in which food outlets were either present or absent [18].

Due to the high number of multiple testing in the analyses, we used Bonferroni correction [23] for P-value to reduce the familywise error rate. The familywise error rate in our testing was α*_fw_* = 1 − (0.95)^9^ * 100 = 36.9% (where the number nine was the number of testing). The adjusted P-value was (0.05/9) = 0.005 to maintain our confidence in our set of analyses. To interpret the results of the regressions, we converted the logged coefficients into practical means following this equation: *exponential* (*coefficient*) − 1.

The statistical software used was Minitab 19, and the corrected *p* ≤ 0.005 was considered statistically significant.

## 3. Results

Table 1 and Table 2 show the general descriptive statistics of socio-economic variables and also the mean BMI, WHR, %fat and age for males and females. The mean number of participants from the UK Biobank in each PD covered by the assessment centres and included in our study was 493. The mean age of the participants was 56.5. The means of BMI, WHR and %fat for men were 27.8 (±4.2), 0.93 (±0.06) and 25.2 (±5.8), respectively. For women, the means of BMI, WHR and %fat were 27.08 (±5.1), 0.81 (±0.07) and 36.5 (±6.9), respectively. Females represented 54.4% and males 45.6% of the total sample. The employed, unemployed and student participants represented 60.4%, 38.3% and 0.3%, respectively. Regarding household income, the highest represented number was for those who earn between £31,000 to £51,999 with 22% of the total sample. Regarding the level of education, people with a university or college degree represented 37%, participants with O levels/GCSEs or equivalent, A levels/AS levels or equivalent, NVQ or HND or HNC or equivalent and CSEs or equivalent represented 20.9%, 11%, 6.5% and 5.4%, respectively. The ethnic groups were divided into Caucasians, Chinese, Indians, blacks, mixed and other. the Caucasians represented 94.1% whilst the rest of the other ethnic groups represented 5.9% of the sample. The mean number of people living in a household was 2.41 with a mean score of -1.29 on Townsend deprivation level. Delivery shops occurred in only 43% of the PDs (Table 3). In contrast, 88% of PDs had FFRs, 86.9% fish and chip shops, 75% takeaways, 100% FSRs, 87.1% pubs, and 93.2% cafes.

**Figure 1:**
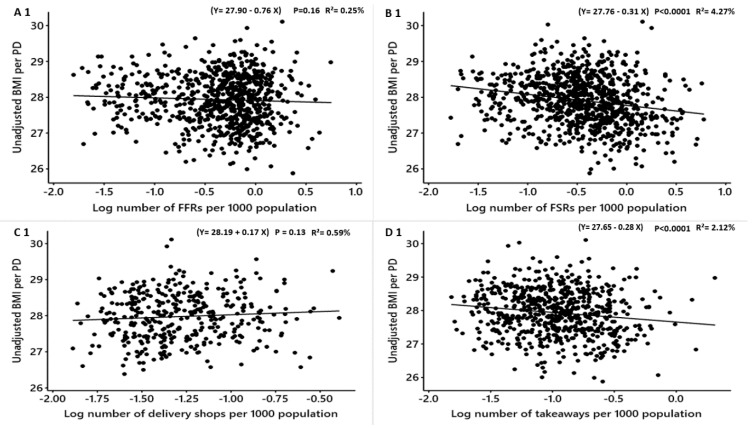

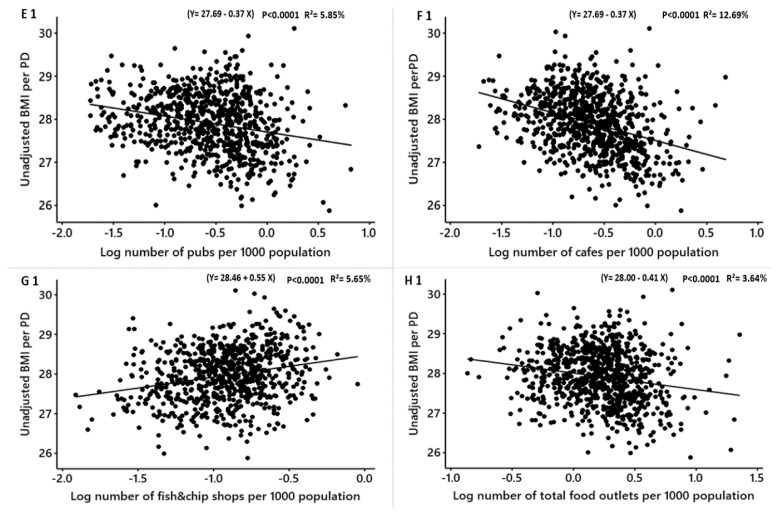

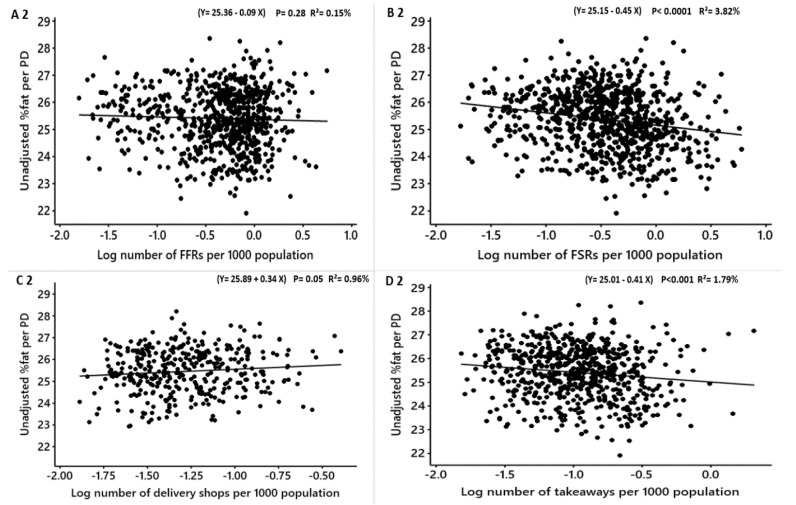

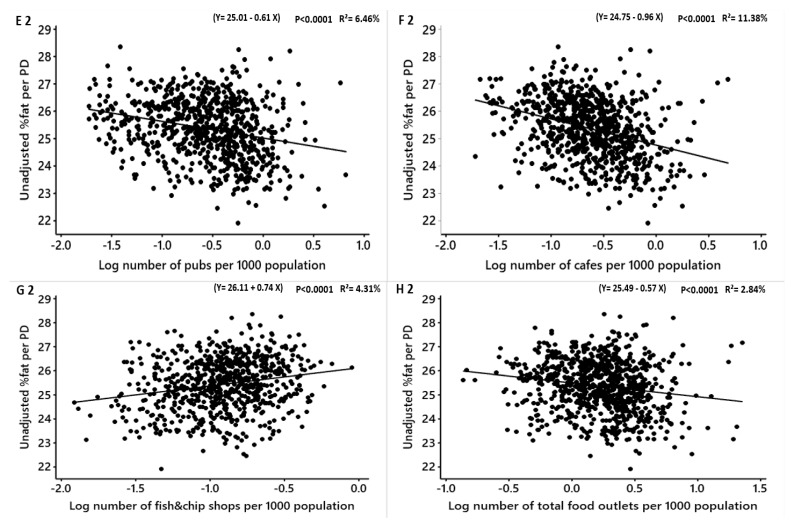

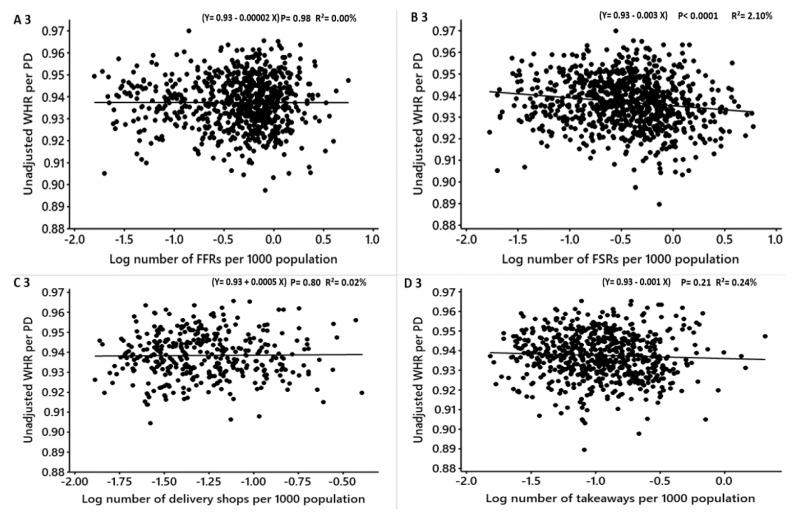

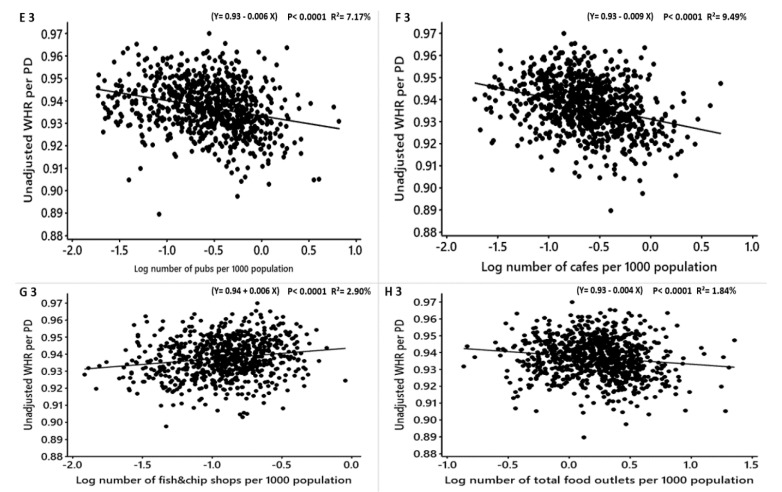
Males: Linear regression analysis of the association between log-transformed numbers of food outlets per 1000 population and unadjusted obesity measure. BMI = Body Mass Index, WHR = Waist to Hip Ratio. FFR = Fast-Food Restaurants, FSR = Full-Service Restaurants, total food outlets = combined.

The associations between the unadjusted obesity measures (BMI, WHR and %fat) for males and different types of food outlets are shown in Table 4. In males, the unadjusted means of BMI, WHR and %fat were significantly positively associated with the density of fish and chip shops (Table 4: BMI: converted β = 0.7, R^2^ = 5.65%, P<0.0001; WHR: converted β = 0.006, R^2^ = 2.90% *p* < 0.0001; %fat: converted β = 1.09 R^2^ = 4.31%, *p* < 0.0001) (Table 4; Figure 1). This means for each increase in the number of fish and chip shops in a PD, the means of the unadjusted BMI, WHR and %fat in males increased by 0.7, 0.006 and 1.09 points, respectively. However, the densities of FSRs, pubs and cafes were all significantly negatively related to the unadjusted means of BMI, WHR and %fat (Table 4; Figure 1). The association between the densities of FFRs and delivery shops and the unadjusted means of BMI, WHR and %fat per PD for males were not significant (Table 4; Figure 1). The density of takeaways was not significantly related to the unadjusted mean WHR and significantly negatively associated with the mean BMI and %fat for males (Table 4; Figure 1), respectively. The density of the total food outlets (combined) was significantly inversely related to the mean unadjusted BMI and %fat for males but not related to the unadjusted mean WHR (Table 4; Figure 1).

**Figure 2:**
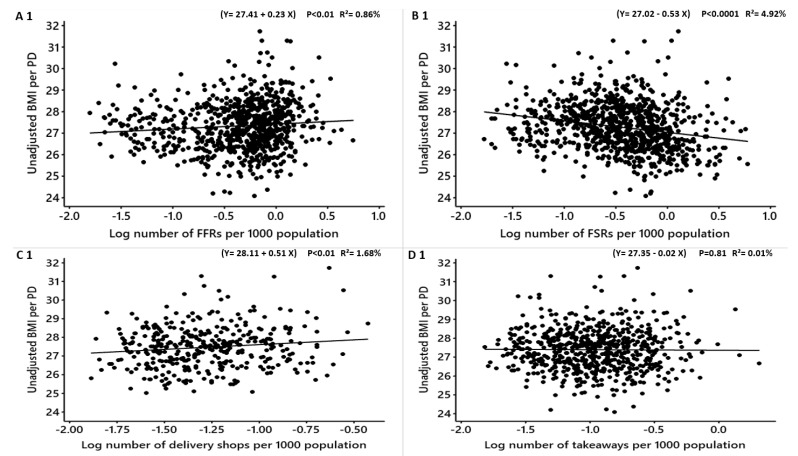

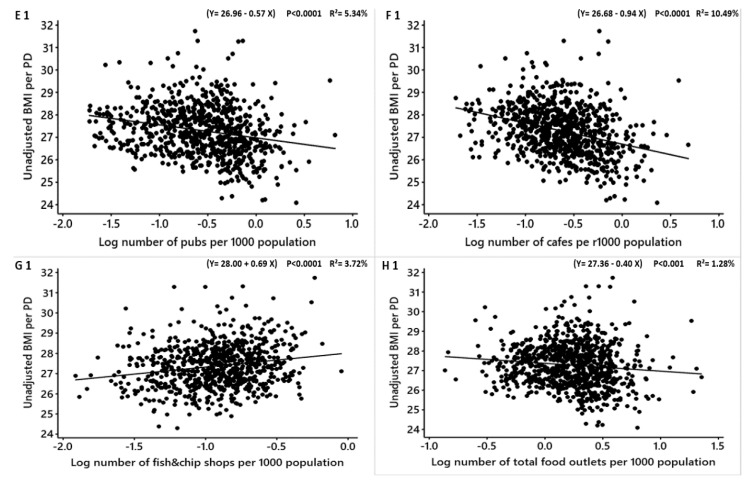

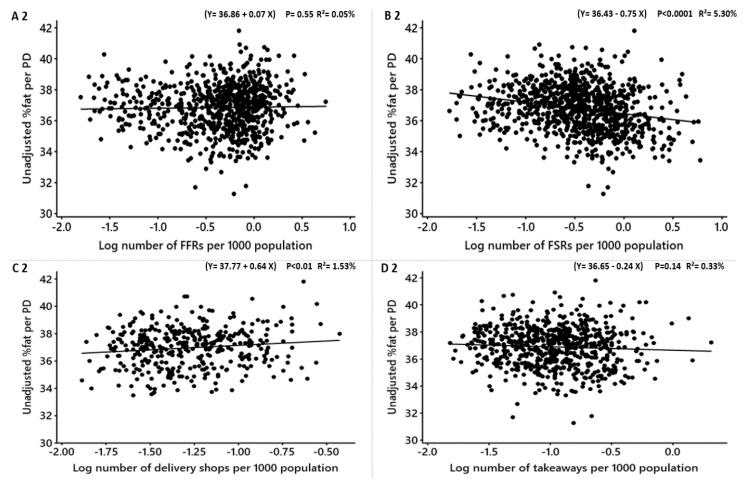

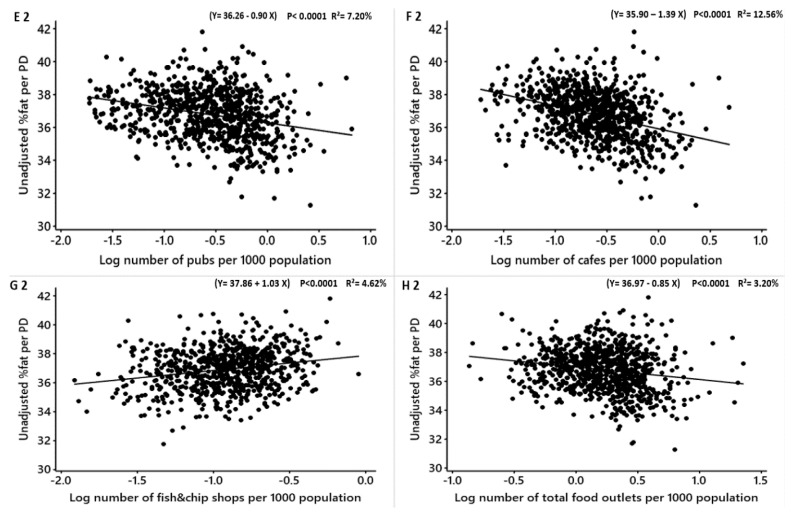

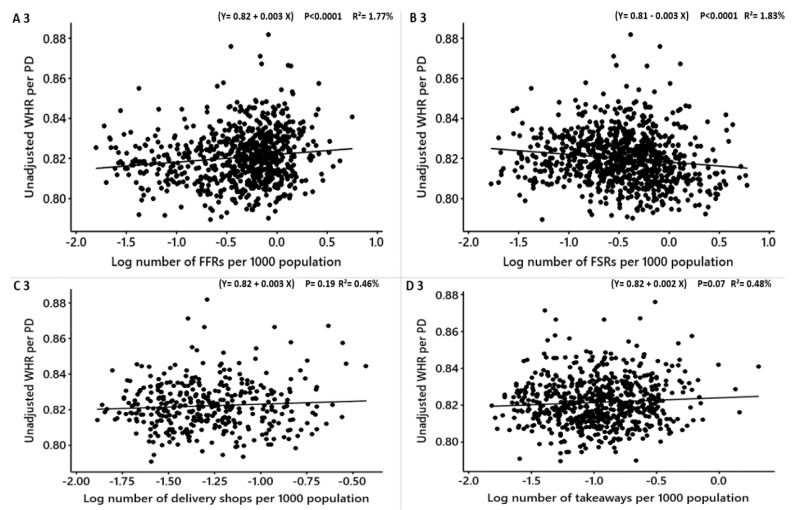

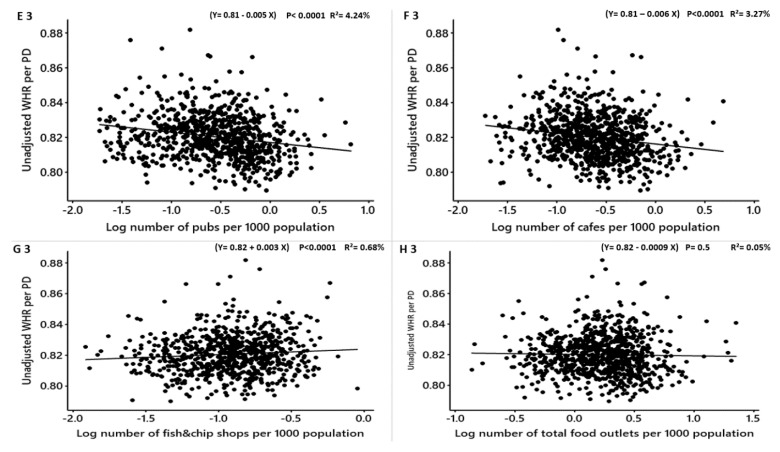
Females: Linear regression analysis of the association between log-transformed numbers of food outlets per 1000 population and unadjusted obesity measures. BMI = Body Mass Index, WHR = Waist to Hip Ratio. FFR = Fast-Food Restaurants, FSR = Full-Service Restaurants, total food outlets = combined.

In females, the associations between the unadjusted obesity measures (BMI, WHR and %fat) and different types of food outlets per PD are shown in Table 5. The density of FFRs was positively significantly associated with WHR but not with the unadjusted BMI and %fat (WHR: converted β = 0.003 R^2^ = 1.77% *p* < 0.0001) (Table 5; Figure 2). For each new FFR opens, the unadjusted WHR may increase among females by 0.003 points on the WHR scale.

The density of delivery shops was positively but not significantly associated with the unadjusted means of BMI, %fat and WHR (Table 5; Figure 2). The unadjusted means of BMI and %fat were significantly positively linked to the density of fish and chip shops but not the mean WHR (BMI: converted β = 0.9 R^2^ = 3.72%, *p* < 0.0001; %fat: converted β = 1.8 R^2^ = 4.62% *p* < 0.0001) (Table 5; Figure 2), respectively. For one fish and chip shop opens in a PD, it may increase the means of the unadjusted BMI and %fat in females by 0.9 and 1.8 points respectively.

The density of takeaways was not related to the obesity measures while FSRs, pubs and cafes were all significantly negatively associated (Table 5; Figure 2). The combined total food outlets density per PD was significantly inversely associated with the unadjusted BMI and %fat and not significantly related to the unadjusted WHR (Table 5; Figure 2).

**Figure 3:**
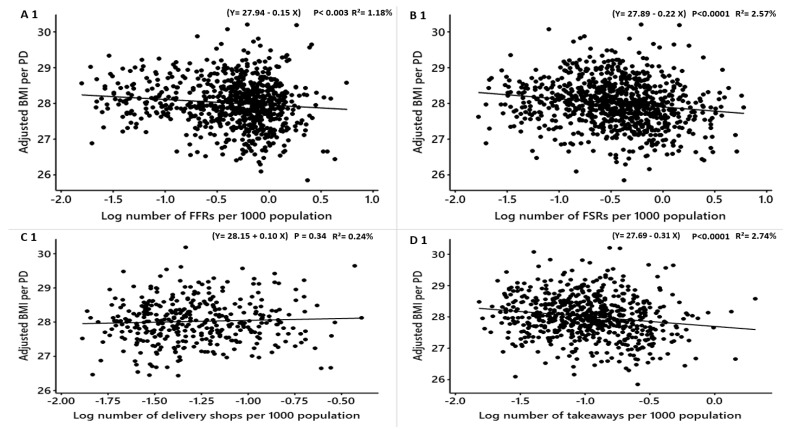

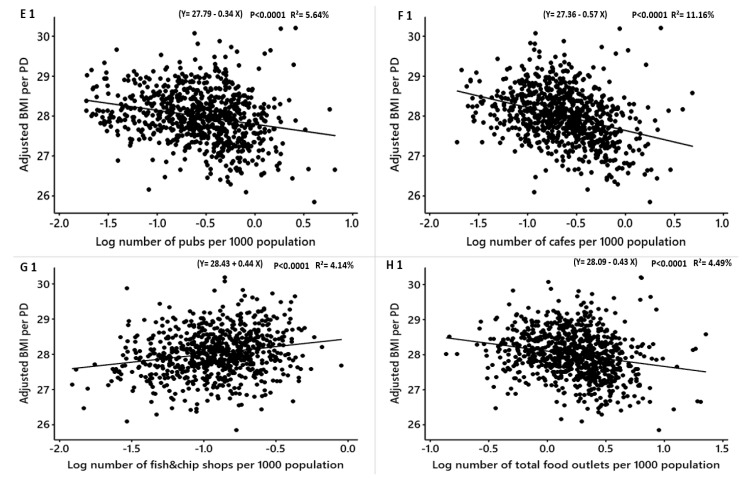

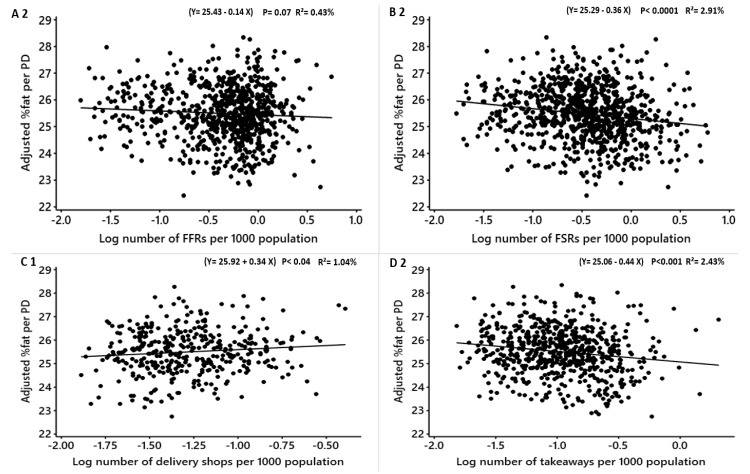

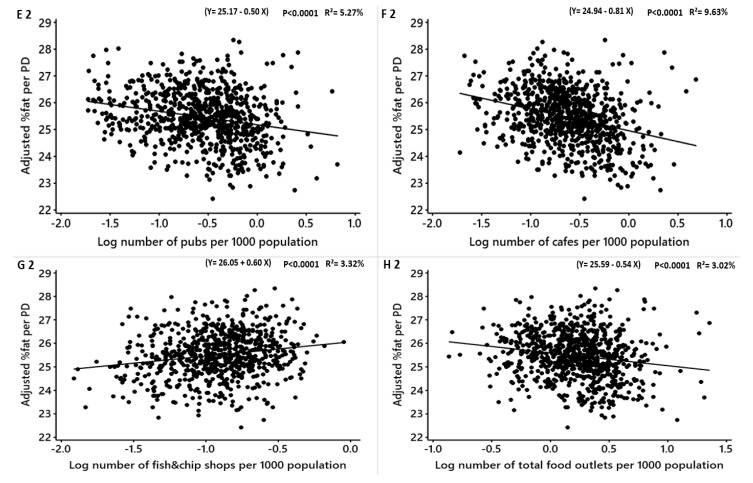

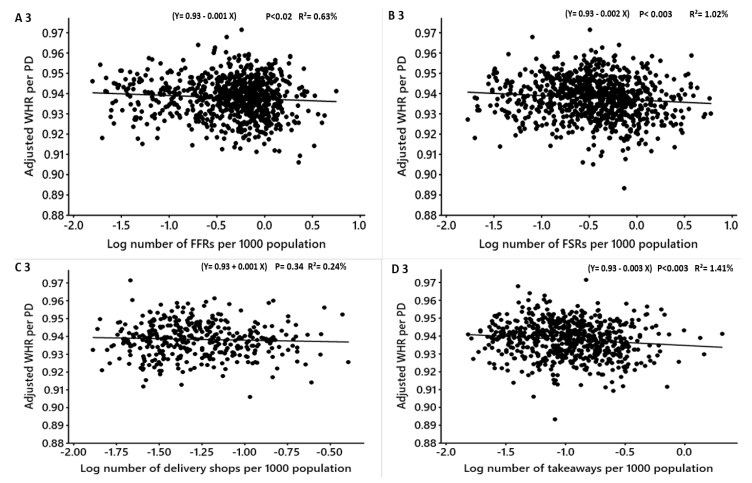

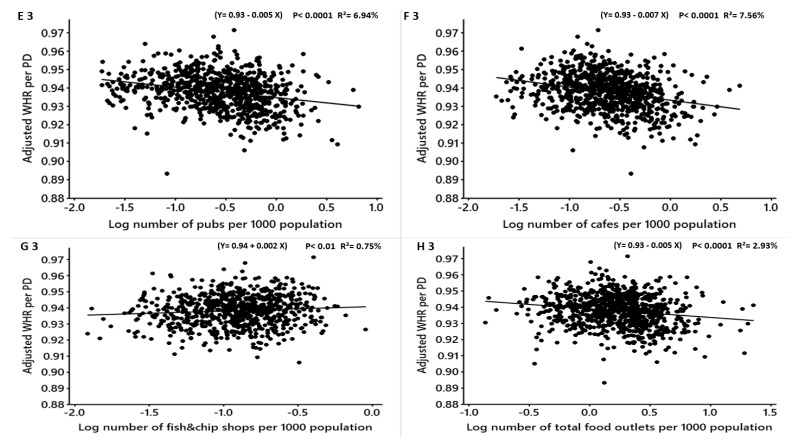
Males: Linear regression analysis of the association between log-transformed numbers of food outlets per 1000 population and adjusted obesity measures. Obesity measures adjusted for deprivation level per household, education, employment, ethnicity, household size, household income and age. BMI = Body Mass Index, WHR = Waist to Hip Ratio. FFR = Fast-Food Restaurants, FSR = Full-Service Restaurants, total food outlets = combined.

The association between the densities of food outlets and obesity measures for males after adjusting for deprivation level, education level, employment, ethnicity, household size, household income and age are shown in Table 6. The density of fish and chip shops was significantly positively related to the adjusted BMI and %fat but not WHR (converted β = 0.5, R^2^ = 4.14%, *p* < 0.0001; converted β = 0.8, R^2^ = 3.32%, *p* < 0.0001) (Table 6; Figure 3), respectively. After converting the coefficients into practical means, for each new fish and chip shop that opens in a PD, the mean BMI and %fat among males may increase by 0.5 and 0.8 points, respectively. The density of delivery shops was not associated with the adjusted BMI, %fat and WHR (Table 6; Figure 3). The densities of takeaways, FSRs, pubs, cafes and total food outlets were significantly negatively associated with the adjusted BMI, the adjusted WHR and the adjusted %fat (Table 6; Figure 3).

**Figure 4:**
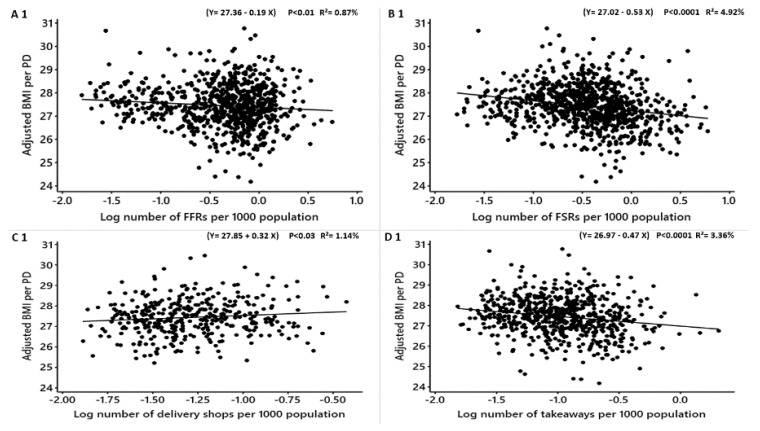

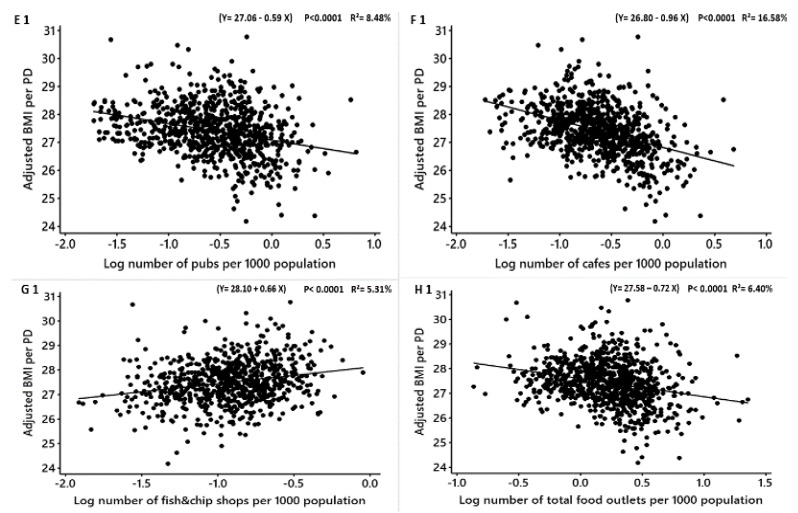

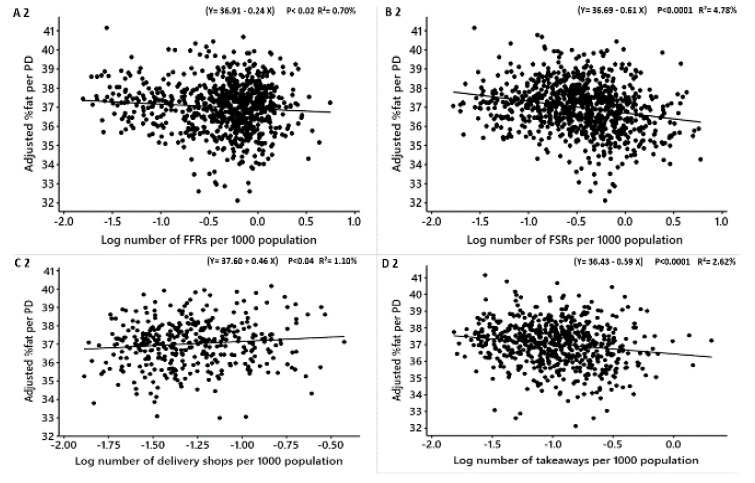

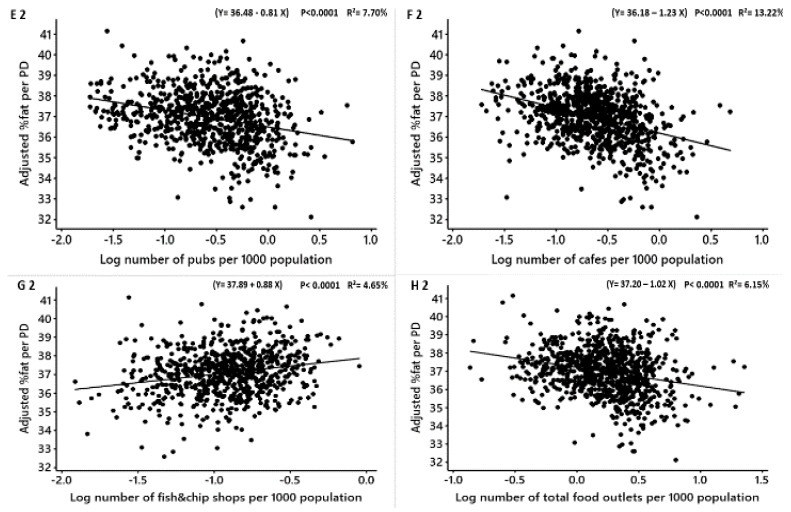

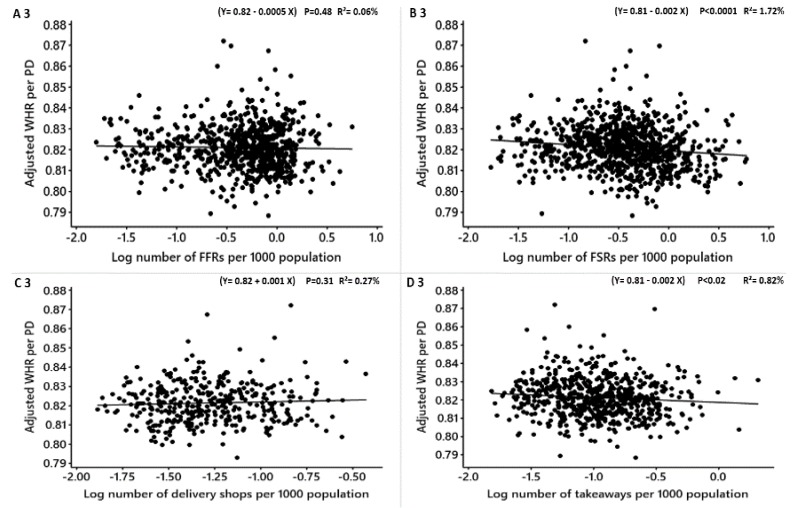

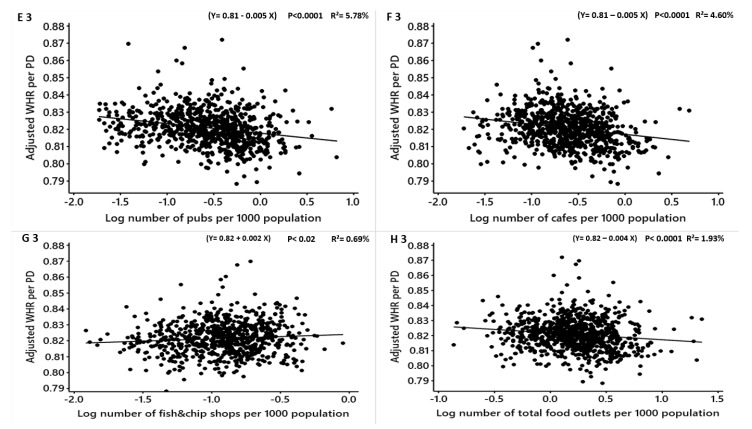
Females: Linear regression analysis of the association between log-transformed numbers of food outlets per 1000 population and adjusted obesity measures. Obesity measures adjusted for deprivation level per household, education, employment, ethnicity, household size, household income and age. BMI = Body Mass Index, WHR = Waist to Hip Ratio. FFR = Fast-Food Restaurants, FSR = Full-Service Restaurants, total food outlets = combined.

The association between the densities of food outlets and the adjusted obesity measures for females are shown in Table 7. The adjusted BMI and %fat, but not WHR, were significantly positively related to the density of fish and chip shops (converted β = 0.9, R^2^ = 5.31%, *p* < 0.0001; converted β = 1.4 R^2^ = 4.65%, *p* < 0.0001) (Table 7; Figure 4: G1, G3 and G2), respectively. This means if one fish and chip shop opens in a PD, BMI and %fat may increase by 0.9 and 1.4 respectively. The density of delivery shops was not related to the adjusted BMI, WHR and %fat (Table 7; Figure 4). The adjusted BMI, WHR and %fat was not significantly related to the density of FFRs (Table 7; Figure 4: A3, A1 and A2). The densities of FSRs, takeaways, pubs, cafes and total food outlets were significantly inversely associated with the adjusted BMI, WHR and %fat (Table 7; Figure 4).

**Figure 5:**
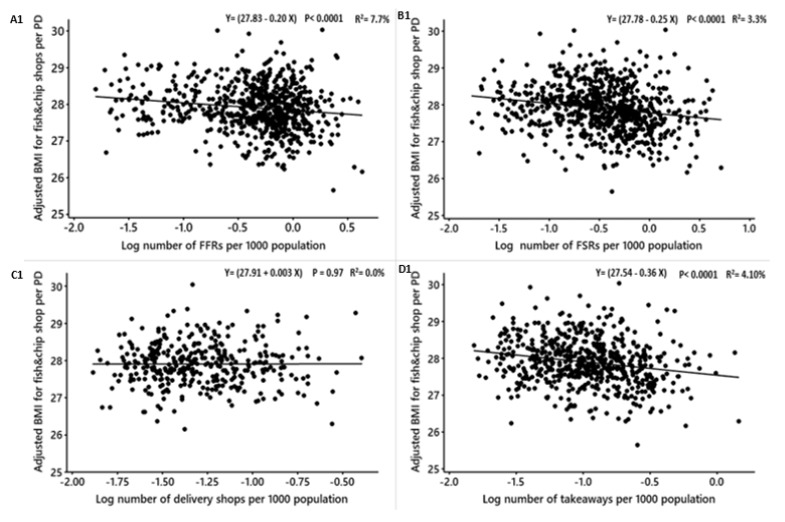

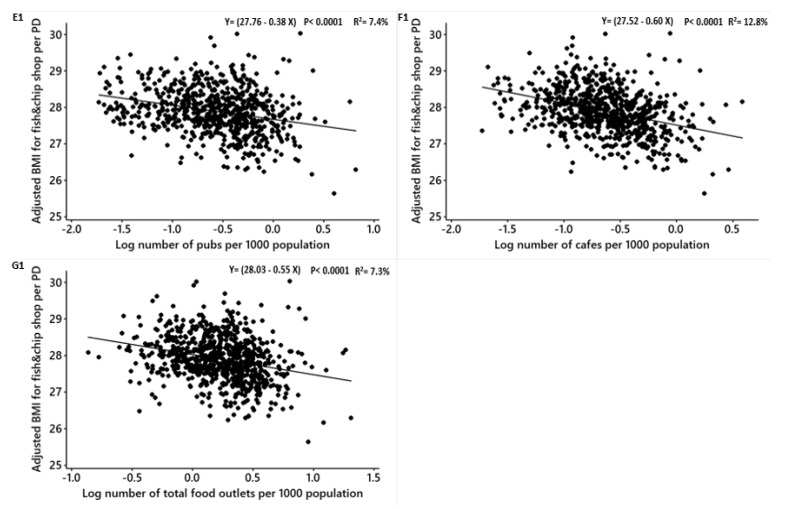

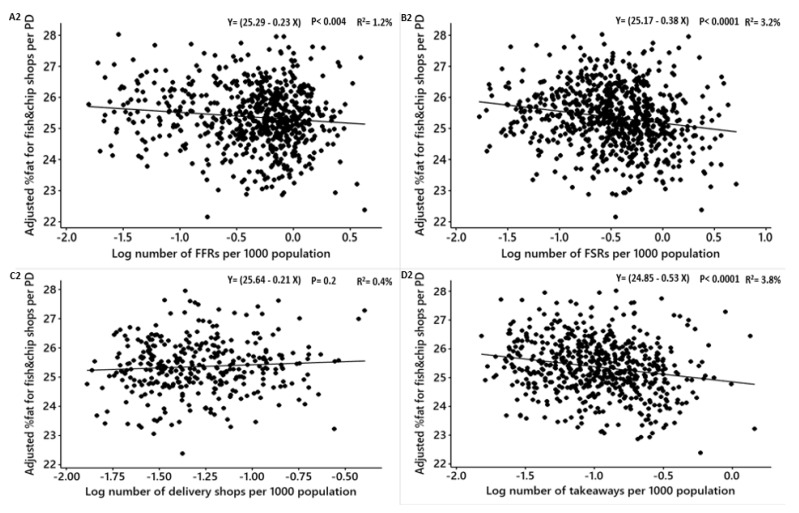

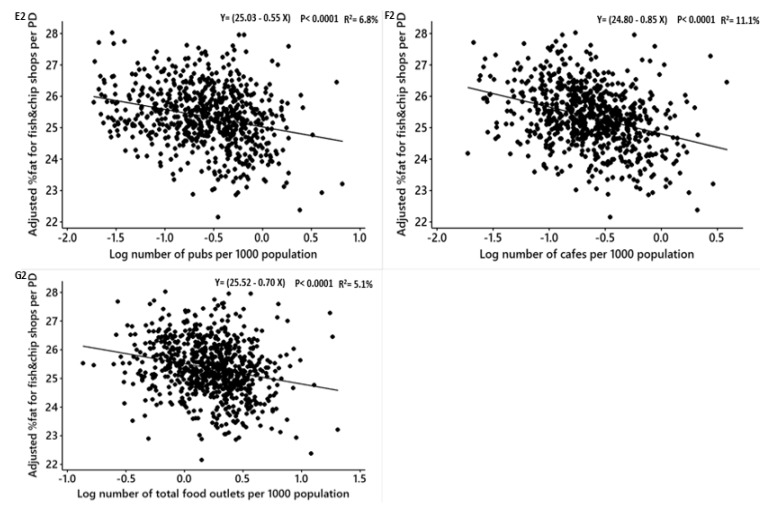

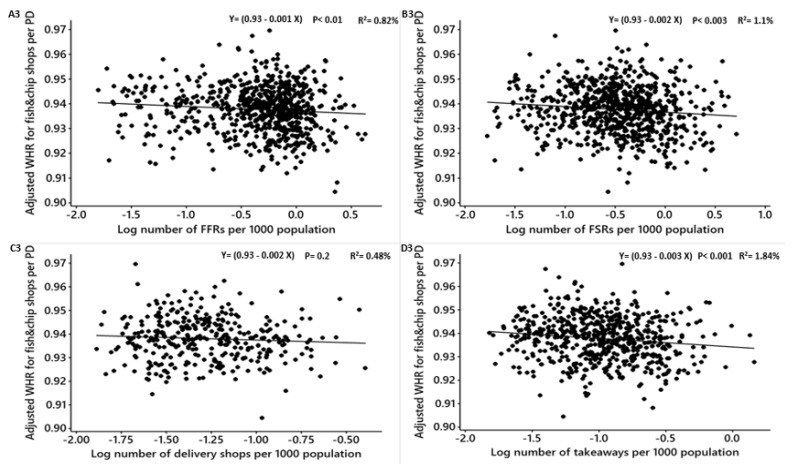

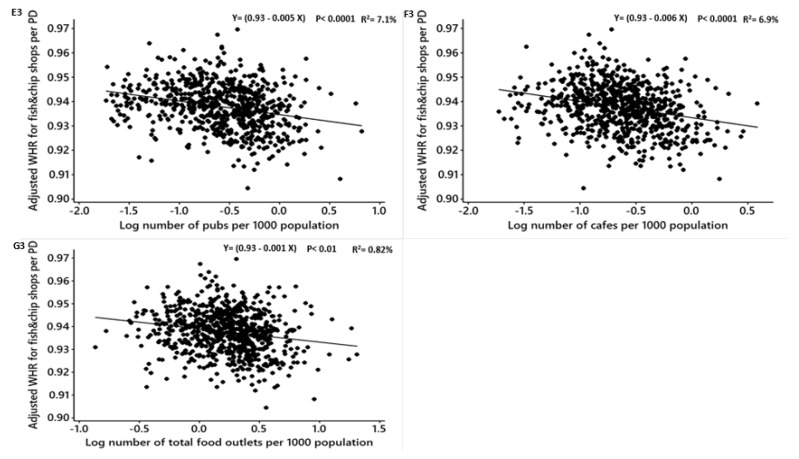
Males: Linear regression analysis of the association between log-transformed numbers of food outlets per 1000 population and adjusted obesity measures. Obesity measures adjusted for deprivation level per household, education, employment, ethnicity, household size, household income, age and density of fish and chip shops. BMI = Body Mass Index, WHR = Waist to Hip Ratio. FFR = Fast-Food Restaurants, FSR = Full-Service Restaurants, total food outlets = combined.

**Figure 6:**
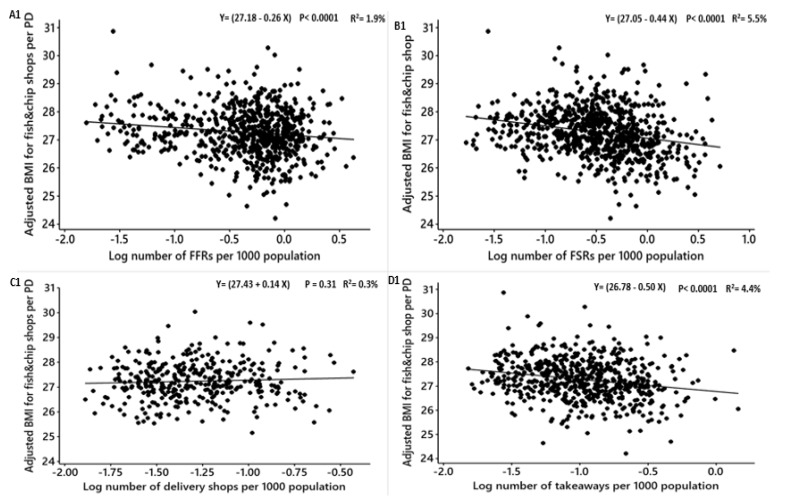

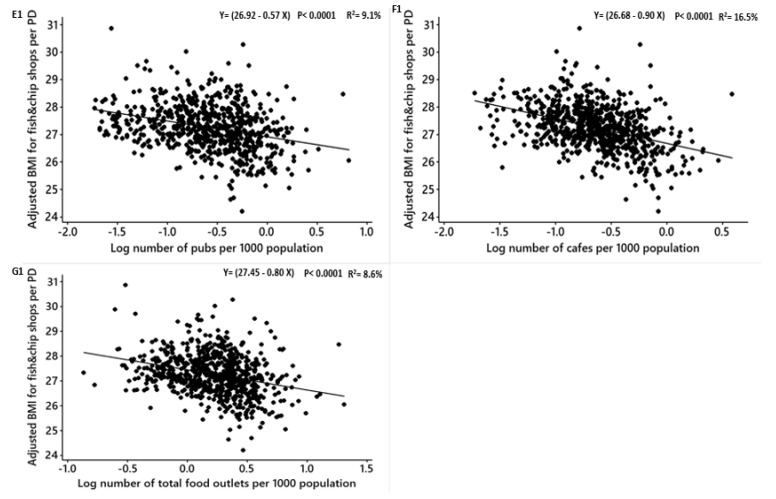

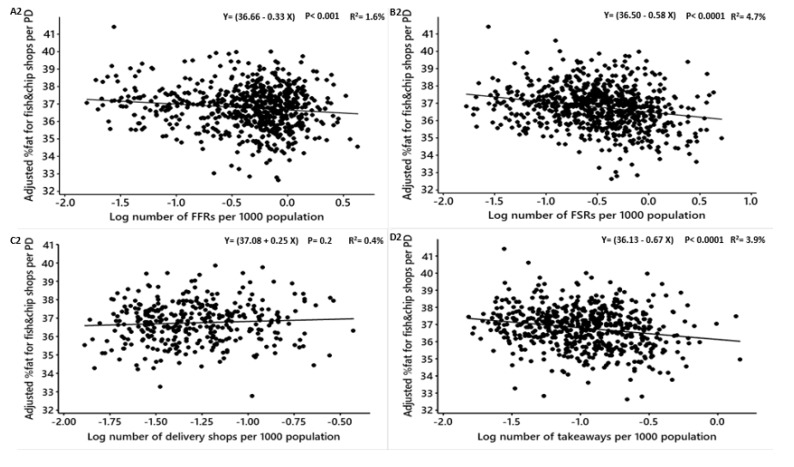

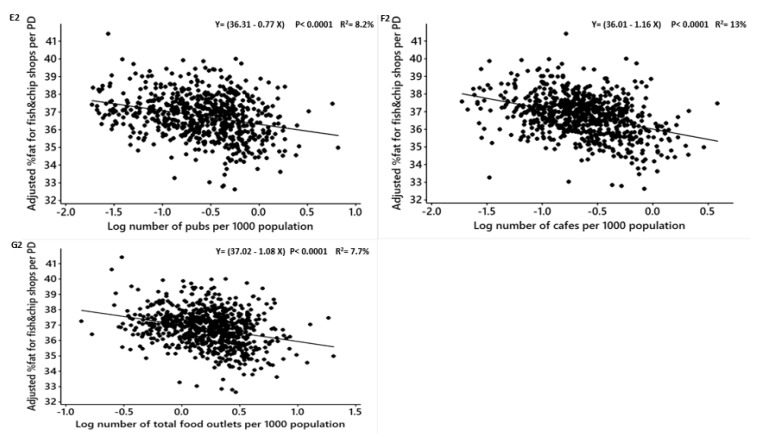

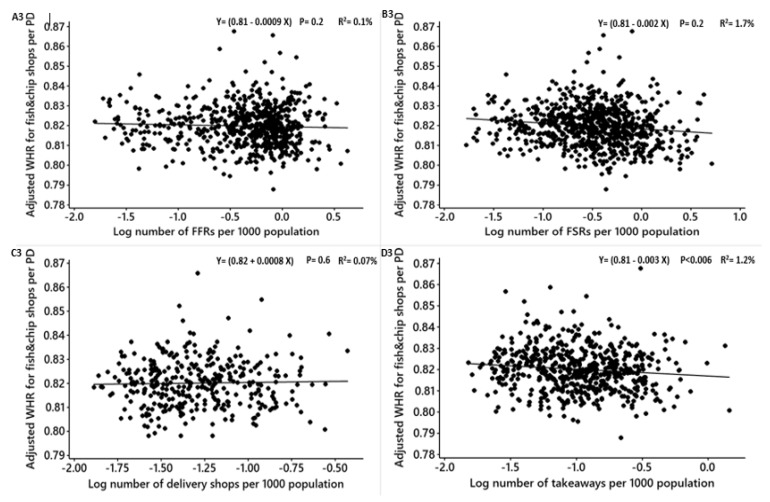

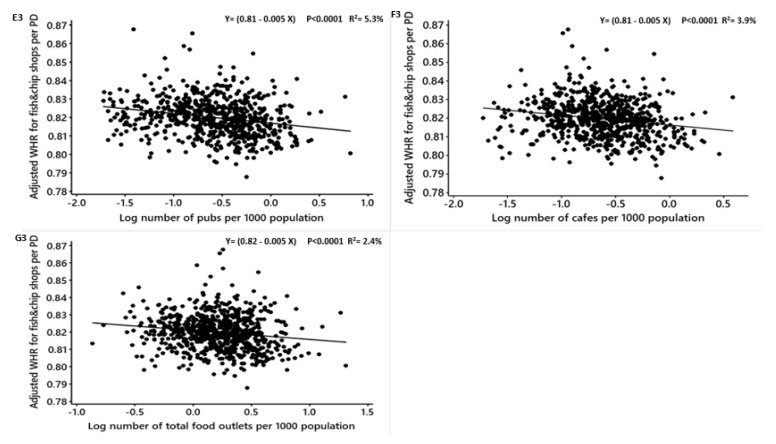
Females: Linear regression analysis of the association between log-transformed numbers of food outlets per 1000 population and adjusted obesity measures. Obesity measures adjusted for deprivation level per household, education, employment, ethnicity, household size, household income, age and density of fish and chip shops. BMI = Body Mass Index, WHR = Waist to Hip Ratio.

We found that the obesity measures in both males and females are associated with the density of fish and chip shops, and to see if fish and chip shops influence the relationships between obesity measures and the other types of food outlets, we further adjusted BMI, WHR and %fat for fish and chip shops beside the socio-economic factors and regressed against the densities of FFRs, FSRs, delivery shops, takeaways, pubs, cafes and total food outlets (Table 8 and Table 9). No associations were noticed between the density of food delivery shops and the obesity measures in both males and females (Table 8. Figure 5; C1, C2, and C3; Table 9. Figure 6; C1, C2, and C3). Also, no association between FFRs density and WHR in females after adjusting for fish and chip shops and socioeconomic factors (Table 9; Figure 6; A3). The densities of FFRs, FSRs, delivery shops, takeaways, pubs, cafes and total food outlets negatively significantly related to the adjusted BMI, %fat and WHR among both males and females (Table 8 and Table 9). No influence was noticed between the association between BMI, %fat and WHR among males and females and FFRs, FSRs, delivery shops, takeaways, pubs, cafes and total food outlets after the further adjustment for fish and chip shops density.

**Figure A1:**
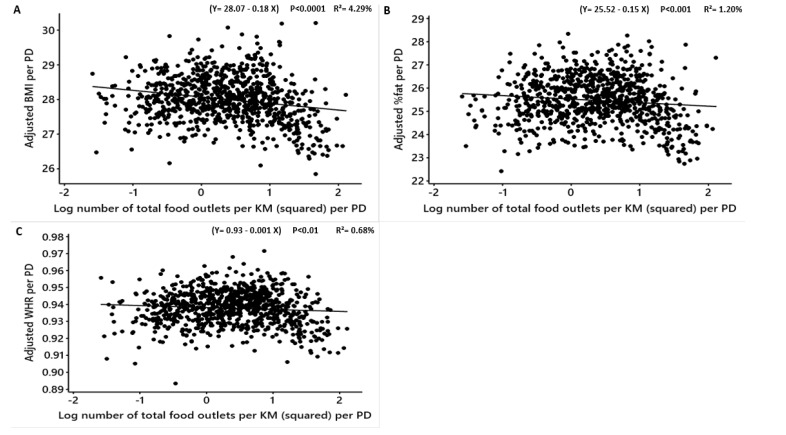
Males: Linear regression analysis of the association between log number of food outlets per KM^2^ per PD and adjusted obesity measures. Obesity measures adjusted for deprivation level per household, education, employment, ethnicity, household size, household income and age. BMI = Body Mass Index, WHR = Waist to Hip Ratio. Total food outlets = number of food outlets combined per kilometre squared per postcode district.

**Figure A2:**
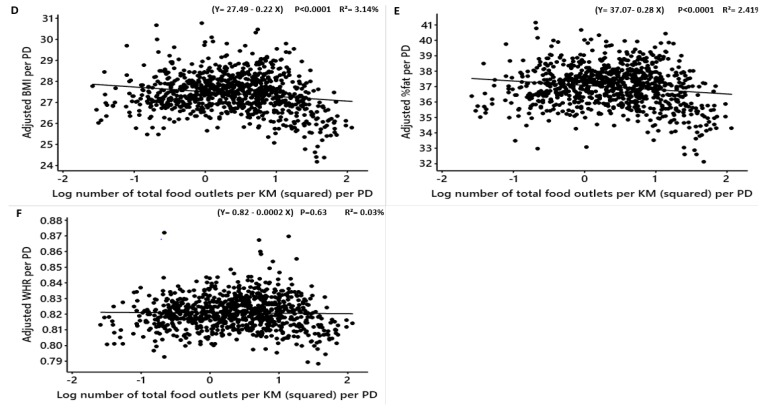
Females: Linear regression analysis of the association between log number of food outlets per KM^2^ per PD and adjusted obesity measures. Obesity measures adjusted for deprivation level per household, education, employment, ethnicity, household size, household income and age. BMI = Body Mass Index, WHR = Waist to Hip Ratio. Total food outlets = number of food outlets combined per kilometre squared per postcode district.

The association between the density of the total food outlets per Square Kilometre (KM^2^) and the mean BMI, %fat and WHR for both sexes after adjustment are shown in the Appendix A (Figure A1 and Figure A2). The associations were significantly negative or disappeared (male adjusted BMI: converted β = −0.16, R^2^ = 4.29%, *p* < 0.0001; male adjusted %fat: converted β = −0.13, R^2^ =1.20%, *p* < 0.0001; male adjusted WHR: converted β = −0.001, R^2^ = 0.68%, *p* = 0.01) (female adjusted BMI: converted β = −0.19, R^2^ = 3.14%, *p* < 0.0001; female adjusted %fat: converted β = −0.24, R^2^ = 2.41%, *p* < 0.0001; female adjusted WHR: converted β = −0.0002, R^2^ = 0.03%, *p* = 0.63) (Figure A1). The adjusted WHR among males and females was not significantly associated with the density of the total food outlets per KM^2^ per PD (male adjusted WHR: converted β = −0.0009, *p* = 0.01; female adjusted WHR: converted β = −0.0002, *p* = 0.63), respectively (Figure A2).

**Figure A3:**
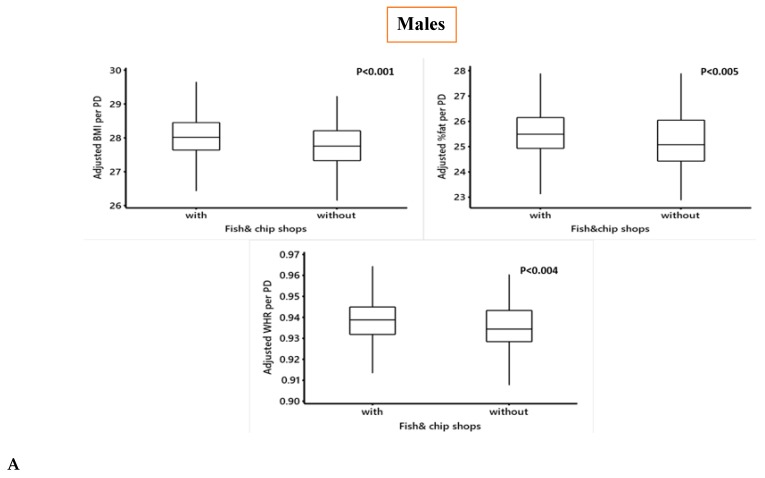

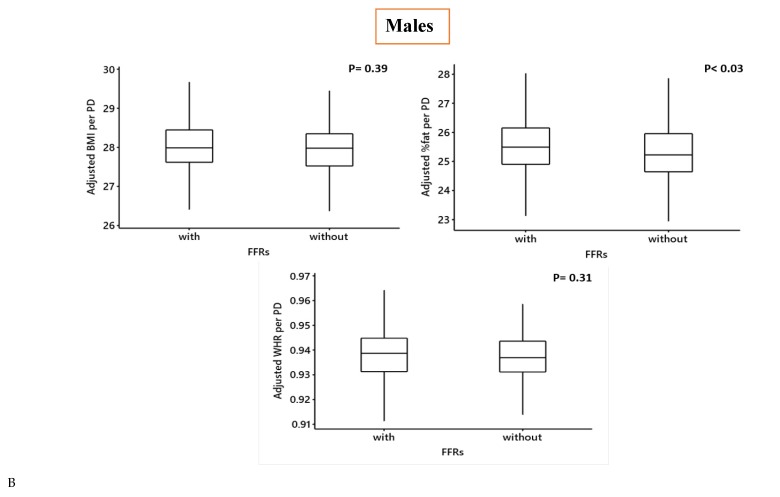

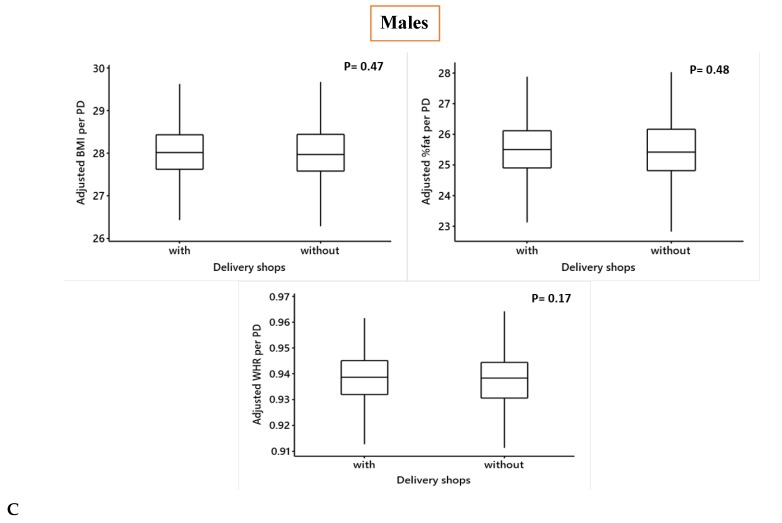

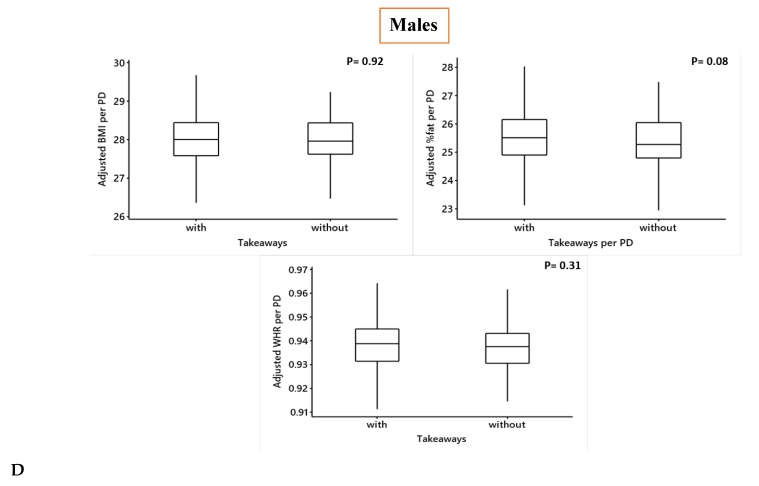

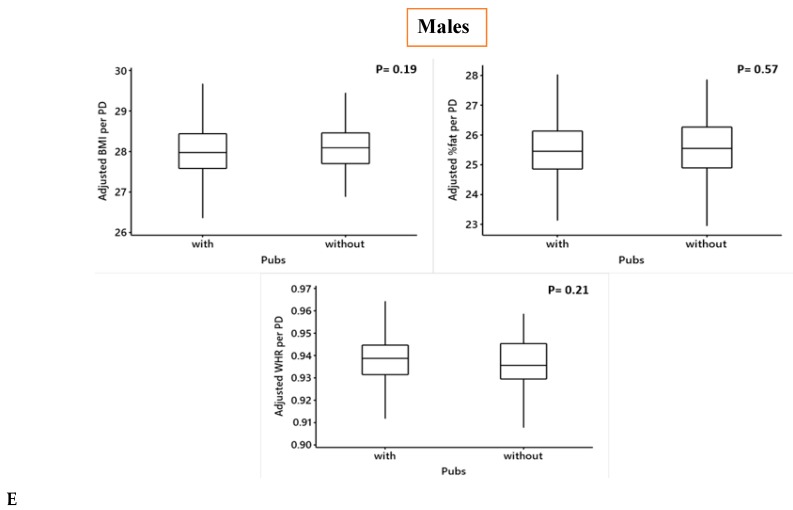

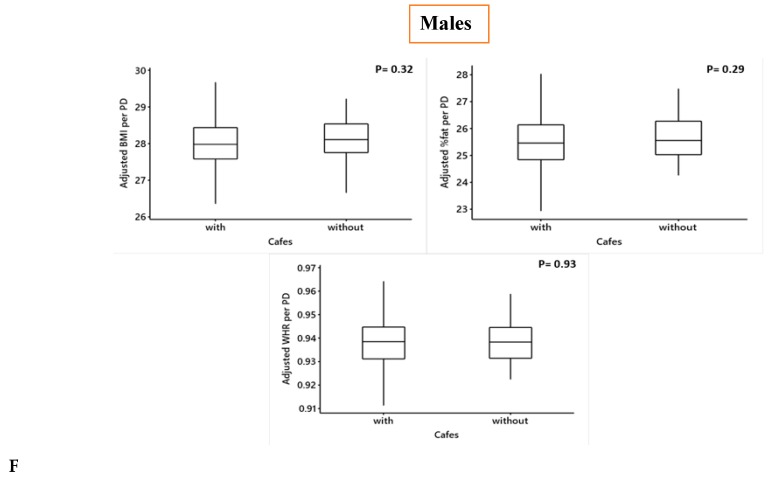

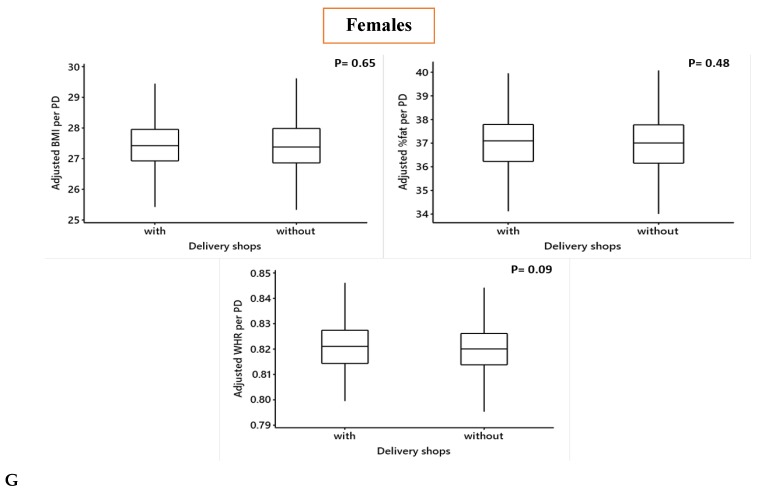

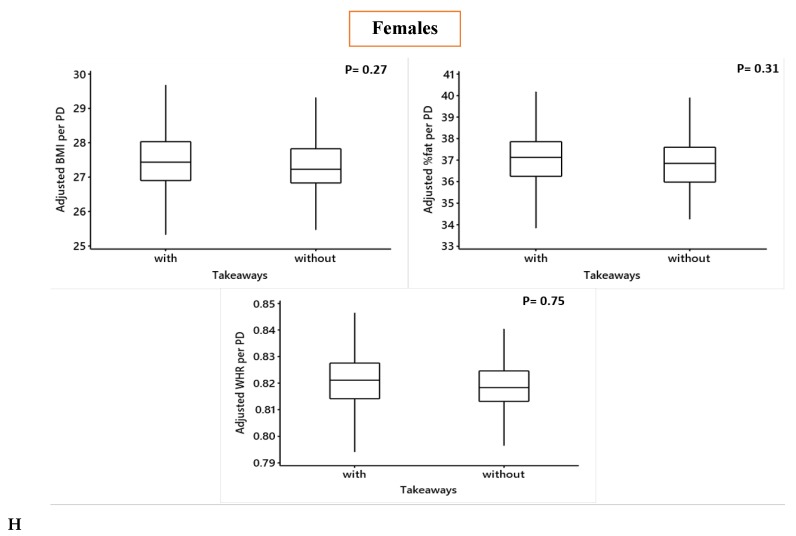

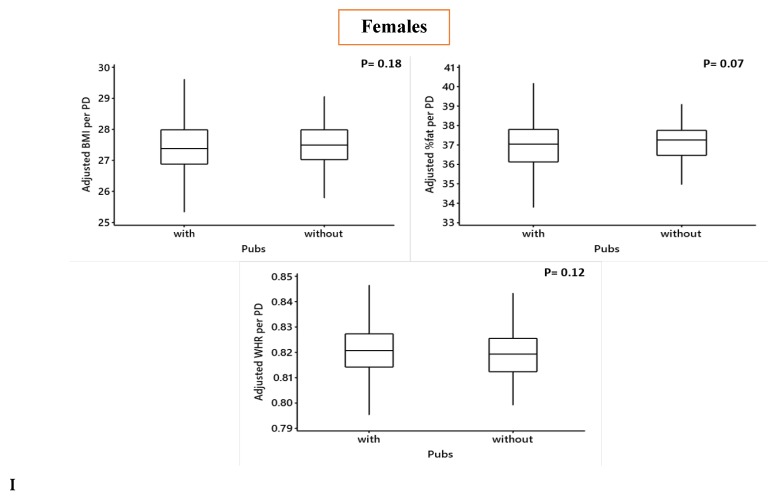

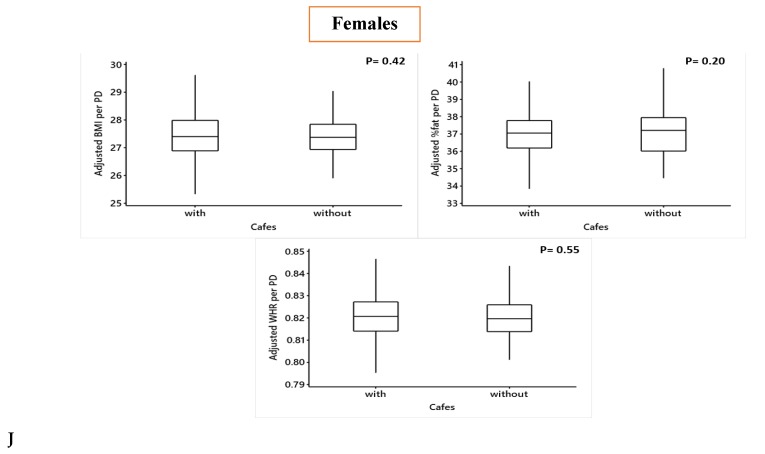
Comparing the adjusted means of BMI, WHR and %fat for males and females in PDs in which some of the given food outlet types were present or absent. Obesity measures adjusted for deprivation level per household, education, employment, ethnicity, household size, household income and age. BMI= Body Mass Index, WHR = Waist to Hip Ratio. Males: **A** = fish & chip shops, **B** = Fast-Food Restaurants, **C** = Delivery shops, **D** = Takeaways, **E** = Pubs, **F** = Cafes; Females: **G** = Delivery shops, **H** = Takeaways, **I** = Pubs, **J** = Cafes.

The adjusted means of BMI, WHR and %fat for both sexes in PDs in which some of the given food outlet types were present or absent are illustrated in Figure A3. Districts with fish and chip shops significantly had higher means of BMI for both sexes (female adjusted BMI: Difference = 0.55, T-value = 5.42, *p* < 0.0001: male adjusted BMI: Difference = 0.24, T-value = 3.46, *p* < 0.001), respectively. Also, the mean %fat in females but not in males was significantly higher in PDs with fish and chip shops (female adjusted % fat: Difference = 0.77, T-value = 5.07, *p* < 0.0001).

The mean adjusted BMI, WHR and %fat for both sexes in PDs where FFRs, delivery, takeaways, pubs and cafes present were not significantly different from the adjusted obesity measures in PDs where these premises absent (Figure A3)

## 4. Discussion

We investigated the association between the densities of different types of food establishments (not supermarkets or groceries) with body mass index (BMI), waist to hip ratio (WHR) and percentage of body fat among 456,079 middle-aged adults registered in the UK Biobank. This study found that fish and chip shops are the only type of food outlet that is significantly positively related to obesity in middle-aged adults in the UK. The obesity measures in both men and women are significantly higher in PDs where fish and chip shops are present in comparison with PDs where these premises are absent. Further, this study shows that the association has a higher impact on women than men. For instance, if one new fish and chip shop opens in a district, females who live in that district may have higher BMI and %fat than males by 0.38 and 0.59 points, respectively. Moreover, no influence was noticed that could affect the associations between the obesity measures in both sexes and different types of food outlets when further adjusted for fish and chip shop density.

On the other hand, inverse or null associations were seen with FFRs, FSRs, delivery, takeaways, pubs, cafes, total food outlets and total food outlets per km^2^. Also, obesity measures for both sexes in districts where some types of food outlets, such as FFRs, delivery, takeaways, pubs and cafes, were present were not different from districts with absent food outlets.

Many possible reasons may explain these mixed results. Jaworowska et al. 2014, found that the mean energy content of fish and chip meals in 64 fish and chip shops across the UK was 1658 calories [24], whilst the mean energy contents of meals in 21 FSRs and 6 FFRs in the UK was 1033 calories and 751 calories [25], respectively. Based on these means of energy contents, one meal of fish and chips on average may have higher energy than a meal from FSRs and FFRs by 37% and 54%, respectively. Second, all fish and chip meals are deep-fried while not all meals in the other types of food outlets are deep-fried. The oil content in deep-fried chips could contain up to 40% of oil, which increases the calorie content [26]. Ansorena 2010, also, illustrated that frying fish whether cod or salmon increases the fat content and energetic value dramatically due to high oil uptake and moisture loss during the process [27]. According to the UK Scientific Advisory Committee on Nutrition, fish and chip meals may contribute up to 80% of a woman’s and 64% of a man’s estimated average daily energy intake [28,29]. Third, fish and chip meals could be more culturally preferable among middle-aged adults when they eat out compared to fast food [30].

In contrast, Mazidi and Speakman noted that the consumption of foods at FFR and FSR restaurants accounts for on average only 15.9% of the total consumption of calories in the U.S [18]. One of the possible reasons for not finding a relationship between some types of food outlets (e.g., FSRs, FFRs, takeaways, pubs and cafes) and obesity measures is because of the low frequency of usage of these food outlets by middle-aged adults. Previous studies have suggested that out-of-home eating decreases with increasing age [14,31]. Finally, the absence of a relationship or indeed in some cases a negative relationship may be because the food provided in these establishments is not any less healthy than, and may even be better than, the normal foods consumed in the home. Hence focussing public policy interventions on reducing food consumption in such establishments may have minimal impact on obesity levels. We suggest that other factors likely have a more important role in the development of obesity and that interventions aimed at curbing intake at public food establishments may have a minimal impact [32,33]. This is potentially because the food supplied at such establishments may not significantly less healthy than what is eaten elsewhere (and may even be better) [34,35]. Policy interventions for obesity should consider the potentially negative role of fish and chip shops in the UK.

## 5. Strengths and Limitations

The weight and height, %fat, waist and hip circumferences were measured by health professionals and not self-reported, which gives accurate measurements. Another major strength is the use of multiple sources of information to accurately quantify the numbers of different food outlets. Other studies focused on only one source. By comparing across sources, we found that relying on a single source (e.g., yellow pages) gives incomplete and potentially biased information. We also included a different variety of food outlets and did not examine the effects of only one type of restaurant such as fast-food restaurants which has been the case in some other studies. In this study, three obesity measures (BMI, WHR and %fat) were used. However, this study was cross-sectional and in common with all such studies has issues in ascribing causality between exposure and outcome. The level at which people currently consume fast food and other meals outside the home is likely not driving the epidemic but that does not mean that substantially higher intakes would not be an issue [18] and may depend on socioeconomic status or inequalities [36].

The weakness of this study is that the UK Biobank data as noted in 2017 by Fry et al. are biased due to “health volunteer selection” [37]. Moreover, Fry et al. 2017 noted that the participants in UK Biobank were older and more deprived than the general population [37].

## 6. Conclusions

We found that fish and chip shops are significantly associated with obesity in middle-aged adults in the UK. For other types of food outlets, similar to studies in the USA, we found no or negative relationships between the densities of these food outlets and the adjusted means of the obesity measures. Paying attention only to fast food and full-service restaurants in intervention policy will likely not be very effective. Policy intervention should consider deep-fried fish and chip shops and other deep-fried food served in such restaurants.

However, since our work was focussed on the population level further research is needed at the individual level to establish if there is an association between the rates at which these food outlets are visited, total energy intake and individual obesity levels in the UK.

## Figures and Tables

**Table 1:** Descriptive characteristics of participants.

	Mean	SD	Percent (%)	Total (N)	Missing
Biobank population	496.5	421.18		502,540	46,461
Sex					
Females			54.4%	248,657	46,461
Males			45.6%	207,422	46,461
Age					
Total mean age	56.5	8.09			
Female	56.3	8			
Male	56.7	8.1			
Employment					
Employed			60.4%	303,346	
Unemployed			38.2%	192,079	
Student			0.3%	1343	
Household income					
18,000–30,999			21.5%	108,179	
31,000–51,999			22.0%	110,776	
52,000–100,000			17.2%	86,272	
>100,000			4.6%	22,932	
<18,000			19.3%	97,208	
Education					
University or college degree			37.2%	186,983	
O levels/GCSEs or equivalent			20.9%	105,200	
A levels/AS levels or equivalent			11%	55,326	
NVQ or HND or HNC or equivalent			6.5%	32,730	
CSEs or equivalent represented			5.4%	26,887	
Ethnicity					
Caucasian			94.1%	472,729	
Chinese			0.3%	1574	
Indian			1.6%	8067	
Black			1.6%	8034	
Mixed			0.4%	1952	
Other			1.5%	7407	
Household size	2.41	1.37			
Deprivation level	−1.29	3.09			

SD = Standard Deviation, N = Number.

**Table 2:** Obesity parameters.

	Mean	SD	Total (N)	Missing
**BMI**			456,079	46,461
Females	27.08	5.17		
Males	27.83	4.22		
**%fat**			456,079	46,461
Females	36.59	6.90		
Males	25.28	5.80		
**WHR**			456,079	46,461
Females	0.81	0.07		
Males	0.93	0.06		

BMI = Body Mass Index, WHR = Waist to Hip Ratio.

**Table 3:** Food outlets.

	Total (N)	PDs Covered (N)	PD Covered (%)
Delivery shops	801	406	43.98
FFRs	17,355	815	88.29
FSRs	13,915	923	100
Fish and chip shops	3617	803	86.9
Takeaways	3466	693	75
Pubs	10,610	804	87
Cafes	9264	861	93.2
Total food outlets	59,028	923	100

*N* = Number, PD = Postcode District, FFRs = Fast Food Restaurants, FSRs = Full-Service Restaurants.

**Table 4:** Males: Linear regression analysis of the association between log-transformed numbers of food outlets per 1000 population and unadjusted obesity measures.

Obesity Measure	Food Outlet Type	β	Converted β	99% CI	R^2^ (%)	*T*	Adjusted *P*-Value	Figure 1
Unadjusted mean BMI	FFRs	−0.07	−0.06	−0.18, −0.03	0.25	−1.38	0.16	A 1
	FSRs	−0.31	−0.26	−0.41, −0.21	4.27	−6.16	<0.0001	B 1
	Delivery shops	0.17	0.18	−0.05, 0.41	0.59	1.49	0.13	C 1
	Takeaways	−0.28	−0.24	−0.44, −0.13	2.12	−3.72	<0.0001	D 1
	Pubs	−0.37	−0.30	−0.48, −0.26	5.85	−6.81	<0.0001	E 1
	Cafes	−0.64	−0.47	−0.76, −0.52	12.69	−10.79	<0.0001	F 1
	Fish and chip shops	0.54	0.71	0.38, 0.70	5.65	6.69	<0.0001	G 1
	Total food outlets (combined)	−0.41	−0.33	−0.55, −0.27	3.64	−5.67	<0.0001	H 1
Unadjusted mean %fat	FFRs	−0.09	−0.08	−0.26, 0.07	0.15	−1.07	0.28	A 2
	FSRs	−0.45	−0.36	−0.61, −0.30	3.82	−5.82	<0.0001	B 2
	Delivery shops	0.34	0.40	−0.01, 0.71	0.96	1.90	0.05	C 2
	Takeaways	−0.41	−0.33	−0.64, −0.17	1.79	−3.42	<0.001	D 2
	Pubs	−0.61	−0.45	−0.77, −0.44	6.46	−7.18	<0.0001	E 2
	Cafes	−0.96	−0.61	−1.14, −0.77	11.38	−10.15	<0.0001	F 2
	Fish and chip shops	0.74	1.09	0.49, 1.0	4.31	5.80	<0.0001	G 2
	Total food outlets (combined)	−0.57	−0.43	−0.79, −0.34	2.84	−4.99	<0.0001	H 2
Unadjusted mean WHR	FFRs	−0.00002	−0.00002	−0.0018, 0.0017	0.00	−0.02	0.98	A 3
	FSRs	−0.003	−0.002	−0.005, −0.001	2.10	−4.27	<0.0001	B 3
	Delivery shops	0.0005	0.0005	−0.003, 0.004	0.02	0.25	0.80	C 3
	Takeaways	−0.001	−0.0009	−0.004, 0.0009	0.24	−1.25	0.21	D 3
	Pubs	−0.006	−0.005	−0.008, −0.005	7.17	−7.60	<0.0001	E 3
	Cafes	−0.009	−0.008	−0.01, −0.007	9.49	−9.17	<0.0001	F 3
	Fish and chip shops	0.007	0.006	0.003, 0.009	2.90	2.29	<0.0001	G 3
	Total food outlets (combined)	−0.004	−0.003	−0.007, −0.002	1.84	−3.99	<0.0001	H 3

BMI = Body Mass Index, WHR = Waist to Hip Ratio, FFRs = Fast Food Restaurants, FSR = Full-Service Restaurants, CI = Confidence Interval.

**Table 5:** Females: Linear regression analysis of the association between log-transformed numbers of food outlets per 1000 population and unadjusted obesity measures.

Obesity Measure	Food Outlet Type	β	Converted β	99% CI	R^2^ (%)	*T*	Adjusted *P*-Value	Figure 2
**Unadjusted mean BMI**	FFRs	0.23	0.25	0.05, 0.40	0.86	2.57	0.01	A 1
	FSRs	−0.53	−0.41	−0.69, −0.37	4.92	−6.65	<0.0001	B 1
	Delivery shops	0.51	0.66	0.11, 0.90	1.68	2.53	0.01	C 1
	Takeaways	−0.02	−0.01	−0.27, 0.21	0.01	−0.23	0.81	D 1
	Pubs	−0.57	−0.43	−0.75, −0.40	5.34	−6.51	<0.0001	E 1
	Cafes	−0.94	−0.60	−1.13, −0.75	10.49	−9.72	<0.0001	F 1
	Fish and chip shops	0.69	0.99	0.44, 0.94	3.72	5.37	<0.0001	G 1
	Total food outlets (combined)	−0.40	−0.32	−0.63, −0.16	1.28	−3.34	<0.001	H 1
**Unadjusted mean %fat**	FFRs	0.07	0.072	−0.16, 0.31	0.05	0.59	0.55	A 2
	FSRs	−0.75	−0.52	−0.96, −0.54	5.30	−6.92	<0.0001	B 2
	Delivery shops	0.64	0.89	0.11, 1.16	1.53	2.41	0.01	C 2
	Takeaways	−0.24	−0.21	−0.57, 0.083	0.33	−1.47	0.14	D 2
	Pubs	−0.90	−0.59	−1.14, −0.67	7.20	−7.64	<0.0001	E 2
	Cafes	−1.39	−0.75	−1.65, −1.14	12.56	−10.76	<0.0001	F 2
	Fish and chip shops	1.03	1.80	0.69, 1.36	4.62	6.02	<0.0001	G 2
	Total food outlets (combined)	−0.85	−0.57	−1.14, −0.54	3.20	−5.32	<0.0001	H 2
**Unadjusted mean WHR**	FFRs	0.004	0.003	0.001, 0.005	1.77	3.71	<0.0001	A 3
	FSRs	−0.003	−0.002	−0.005, −0.001	1.83	−4.00	<0.0001	B 3
	Delivery shops	0.0032	0.003	−0.001, 0.008	0.46	1.31	0.19	C 3
	Takeaways	0.003	0.002	−0.0002, 0.005	0.48	1.77	0.07	D 3
	Pubs	−0.005	−0.004	−0.008, −0.003	4.24	−5.77	<0.0001	E 3
	Cafes	−0.006	−0.005	−0.008, −0.003	3.27	−5.22	<0.0001	F 3
	Fish and chip shops	0.004	0.003	0.0004, 0.006	0.68	2.26	0.02	G 3
	Total food outlets (combined)	−0.0009	−0.0008	−0.003, 0.001	0.05	−0.68	0.50	H 3

BMI = Body Mass Index, WHR = Waist to Hip Ratio, FFRs = Fast Food Restaurants, FSR = Full-Service Restaurants, CI = Confidence Interval.

**Table 6:** Males: Linear regression analysis of the association between log-transformed numbers of food outlets per 1000 population and adjusted obesity measures.

Obesity Measure	Food Outlet Type	β	Converted β	99% CI	R^2^ (%)	*t*	Adjusted *P*-Value	Figure 3
Adjusted mean BMI	FFRs	−0.15	−0.13	−0.26, −0.05	1.18	−3.01	<0.003	A 1
	FSRs	−0.22	−0.19	−0.32, −0.13	2.57	−4.74	<0.0001	B 1
	Delivery shops	0.10	0.105	−0.11, 0.33	0.24	0.95	0.34	C 1
	Takeaways	−0.31	−0.26	−0.46, −0.17	2.74	−4.25	<0.0001	D 1
	Pubs	−0.34	−0.28	−0.45, −0.24	5.64	−6.68	<0.0001	E 1
	Cafes	−0.57	−0.43	−0.69, −0.46	11.16	−10.04	<0.0001	F 1
	Fish and chip shops	0.44	0.55	0.28, 0.59	4.14	5.68	<0.0001	G 1
	Total food outlets (combined)	−0.43	−0.34	−0.57, −0.30	4.49	−6.33	<0.0001	H 1
Adjusted mean %fat	FFRs	−0.14	−0.13	−0.30, 0.01	0.43	−1.82	0.07	A 2
	FSRs	−0.36	−0.30	−0.51, −0.22	2.91	−5.06	<0.0001	B 2
	Delivery shops	0.34	0.40	0.002, 0.67	1.04	1.98	0.04	C 2
	Takeaways	−0.44	−0.35	−0.66, −0.22	2.43	−4.00	<0.0001	D 2
	Pubs	−0.50	−0.39	−0.66, −0.35	5.27	−6.45	<0.0001	E 2
	Cafes	−0.81	−0.55	−0.98, −0.64	9.63	−9.24	<0.0001	F 2
	Fish and chip shops	0.60	0.82	0.36, 0.83	3.32	5.07	<0.0001	G 2
	Total food outlets (combined)	−0.54	−0.41	−0.75, −0.33	3.02	−5.15	<0.0001	H 2
Adjusted mean WHR	FFRs	−0.001	−0.0009	−0.003, −0.0001	0.63	−2.19	0.02	A 3
	FSRs	−0.002	−0.001	−0.003, −0.0007	1.02	−2.96	<0.003	B 3
	Delivery shops	−0.001	−0.0009	−0.005, 0.001	0.24	−0.95	0.34	C 3
	Takeaways	−0.003	−0.002	−0.005, −0.001	1.41	−3.03	<0.003	D 3
	Pubs	−0.005	−0.004	−0.007, −0.004	6.94	−7.46	<0.0001	E 3
	Cafes	−0.007	−0.006	−0.008, −0.005	7.56	−8.10	<0.0001	F 3
	Fish and chip shops	0.003	0.002	0.0004, 0.005	0.75	2.38	0.01	G 3
	Total food outlets (combined)	−0.005	−0.004	−0.007, −0.003	2.93	−5.07	<0.0001	H 3

Obesity measures adjusted for deprivation level per household, education, employment, ethnicity, household size, household income and age. BMI = Body Mass Index, WHR = Waist to Hip Ratio, FFRs = Fast Food Restaurants, FSR = Full-Service Restaurants.

**Table 7:** Females: Linear regression analysis of the association between log-transformed numbers of food outlets per 1000 population and adjusted obesity measures.

Obesity Measure	Food Outlet Type	β	Converted β	99% CI	R^2^ (%)	*t*	Adjusted *P*-Value	Figure 4
Adjusted mean BMI	FFRs	−0.19	−0.17	−0.33, −0.04	0.87	−2.59	0.01	A 1
	FSRs	−0.42	−0.34	−0.55, −0.29	4.73	−6.52	<0.0001	B 1
	Delivery shops	0.32	0.37	0.01, 0.63	1.14	2.08	0.03	C 1
	Takeaways	−0.47	−0.37	−0.67, −0.27	3.36	−4.73	<0.0001	D 1
	Pubs	−0.59	−0.44	−0.73, −0.45	8.48	−8.35	<0.0001	E 1
	Cafes	−0.96	−0.61	−1.11, −0.81	16.5	−12.66	<0.0001	F 1
	Fish and chip shops	0.66	0.93	0.44, 0.87	5.31	6.47	<0.0001	G 1
	Total food outlets (combined)	−0.72	−0.51	−0.91, −0.54	6.40	−7.65	<0.0001	H 1
Adjusted mean %fat	FFRs	−0.24	−0.21	−0.45, −0.03	0.70	−2.32	0.02	A 2
	FSRs	−0.61	−0.45	−0.80, −0.43	4.78	−6.56	<0.0001	B 2
	Delivery shops	0.46	0.58	0.01, 0.90	1.10	2.04	0.04	C 2
	Takeaways	−0.59	−0.44	−0.87, −0.31	2.62	−4.16	<0.0001	D 2
	Pubs	−0.81	−0.55	−1.01, −0.61	7.70	−7.92	<0.0001	E 2
	Cafes	−1.23	−0.70	−1.45, −1.01	13.22	−11.08	<0.0001	F 2
	Fish and chip shops	0.88	1.41	0.59, 1.17	4.65	6.04	<0.0001	G 2
	Total food outlets (combined)	−1.02	−0.63	−1.29, −0.75	6.15	−7.49	<0.0001	H 2
Adjusted mean WHR	FFRs	−0.0005	−0.0004	−0.002, 0.001	0.06	−0.70	0.48	A 3
	FSRs	−0.002	−0.001	−0.004, −0.001	1.72	−3.87	<0.0001	B 3
	Delivery shops	0.001	0.001	−0.001, 0.005	0.27	1.00	0.31	C 3
	Takeaways	−0.002	−0.001	−0.005, −0.0004	0.82	−2.31	0.02	D 3
	Pubs	−0.006	−0.0059	−0.007, −0.003	5.78	−6.79	<0.0001	E 3
	Cafes	−0.0059	−0.0058	−0.007, −0.004	4.60	−6.23	<0.0001	F 3
	Fish and chip shops	0.003	0.002	0.0003, 0.005	0.69	2.27	0.02	G 3
	Total food outlets (combined)	−0.004	−0.003	−0.006, −0.002	1.93	−4.10	<0.0001	H 3

Obesity measures adjusted for deprivation level per household, education, employment, ethnicity, household size, household income and age. BMI = Body Mass Index, WHR = Waist to Hip Ratio, FFRs = Fast Food Restaurants, FSR = Full-Service Restaurants, CI = Confidence Interval.

**Table 8:** Males: Linear regression analysis of the association between log-transformed numbers of food outlets per 1000 population and obesity measures adjusted for deprivation level per household, education, employment, ethnicity, household size, household income, age and fish and chip shops.

Obesity Measure Adjusted for Fish and Chip Shops	Type of Food Outlet	β	Converted β	99% CI	R^2^ (%)	*t*	Adjusted *P*-Value	Figure 5
Adjusted mean BMI	FFRs	−0.20	−0.181	−0.31, −0.10	2.2	−3.94	0.0001	A1
	FSRs	−0.2	−0.181	−0.35, −0.15	3.3	−5.11	0.0001	B1
	Delivery	0.003	0.0030	−0.21, 0.21	0	0.03	0.9	C1
	Takeaways	−0.3	−0.25	−0.50, −0.22	4.1	−5.02	0.0001	D1
	Pubs	−0.3	−0.25	−0.49, −0.28	7.4	−7.30	0.0001	E1
	Cafes	−0.6	−0.45	−0.71, −0.48	12.8	−10.28	0.0001	F1
	Total food outlets (combined)	−0.5	−0.39	−0.69, −0.41	7.3	−7.67	0.0001	G1
Adjusted mean %fat	FFRs	−0.2	−0.18	−0.39, −0.07	1.2	−2.88	0.004	A2
	FSRs	−0.3	−0.25	−0.54, −0.23	3.2	−5.03	0.0001	B2
	Delivery	0.2	0.22	−0.11, 0.55	0.4	1.27	0.2	C2
	Takeaways	−0.5	−0.39	−0.74, −0.31	3.8	−4.84	0.0001	D2
	Pubs	−0.5	−0.39	−0.71, −0.40	6.8	−7.00	0.0001	E2
	Cafes	−0.8	−0.55	−1.03, −0.67	11.1	−9.48	0.0001	F2
	Total food outlets (combined)	−0.7	−0.50	−0.92, −0.49	5.1	−6.37	0.0001	G2
Adjusted mean WHR	FFRs	−0.001	−0.0009	−0.0034, −0.00032	0.82	−2.37	0.01	A3
	FSRs	−0.002	−0.0019	−0.0034, −0.0007	1.17	−2.98	0.003	B3
	Delivery	0.002	0.0020	−0.005, 0.001	0.48	−1.28	0.20	C3
	Takeaways	−0.003	−0.0029	−0.005, −0.0001	1.84	−3.32	0.001	D3
	Pubs	−0.005	−0.0049	−0.007, −0.004	7.1	−7.13	0.0001	E3
	Cafes	−0.006	−0.0059	−0.008, −0.004	6.9	−7.33	0.0001	F3
	Total food outlets (combined)	−0.005	−0.0049	−0.007, −0.003	3.6	−5.29	0.0001	G3

BMI = Body Mass Index, WHR = Waist to Hip Ratio, FFRs = Fast Food Restaurants, FSR = Full-Service Restaurants, CI = Confidence Interval.

**Table 9:** Females: Linear regression analysis of the association between log-transformed numbers of food outlets per 1000 population and obesity measures adjusted for deprivation level per household, education, employment, ethnicity, household size, household income, age and fish and chip shops.

Obesity Measure Adjusted for Fish and Chip Shops	Type of Food Outlet	β	Converted β	99% CI	R^2^ (%)	*t*	Adjusted *P*-Value	Figure 6
Adjusted mean BMI	FFRs	−0.26	−0.22	−0.44, −0.07	1.9	−3.6	0.0001	A1
	FSRs	−0.44	−0.32	−0.61, −0.26	5.5	−6.6	0.0001	B1
	Delivery	0.14	0.15	−0.23, 0.52	0.30	1.01	0.3	C1
	Takeaways	−0.5	−0.39	−0.75, −0.25	4.4	−5.25	0.0001	D1
	Pubs	−0.5	−0.39	−0.33, −0.04	9.1	−8.20	0.0001	E1
	Cafes	−0.9	−0.59	−1.09, −0.70	16.5	−11.92	0.0001	F1
	Total food outlets (combined)	−0.8	−0.55	−0.1.05, −0.55	8.6	−8.42	0.0001	G1
Adjusted mean %fat	FFRs	−0.3	−0.25	−0.60, −0.07	1.6	−3.33	0.001	A2
	FSRs	−0.5	−0.39	−0.8, −0.33	4.7	−6.12	0.0001	B2
	Delivery	0.2	0.22	−0.28, −0.80	0.4	1.22	0.2	C2
	Takeaways	−0.6	−0.45	−1.03, −0.32	3.9	−4.93	0.0001	D2
	Pubs	−0.7	−0.50	−1.03, −0.51	8.7	−7.71	0.0001	E2
	Cafes	−1.1	−0.66	−1.44, −0.87	13.4	−10.58	0.0001	F2
	Total food outlets (combined)	−1.08	−0.66	−1.44, 0.73	7.7	−7.93	0.0001	G2
Adjusted mean WHR	FFRs	−0.0009	−0.0009	−0.003, −0.001	0.17	−1.08	0.28	A3
	FSRs	−0.002	−0.0019	−0.005, −0.0008	1.75	−3.65	0.0001	B3
	Delivery	0.0008		−0.003, 0.005	0.07	0.47	0.63	C3
	Takeaways	−0.003	−0.0029	−0.006, 0.0001	1.26	−2.74	0.006	D3
	Pubs	−0.005	−0.0049	−0.007, −0.003	5.33	−6.12	0.0001	E3
	Cafes	−0.005	−0.0049	−0.007, −0.002	3.91	−5.40	0.0001	F3
	Total food outlets (combined)	−0.005	−0.0049	−0.008, −0.002	2.4	−4.31	0.0001	G3

BMI = Body Mass Index, WHR = Waist to Hip Ratio, FFRs = Fast Food Restaurants, FSR = Full-Service Restaurants, CI = Confidence Interval.
